# LncRNA MTX2-6 Suppresses Cell Proliferation by Acting as ceRNA of miR-574-5p to Accumulate SMAD4 in Esophageal Squamous Cell Carcinoma

**DOI:** 10.3389/fcell.2021.654746

**Published:** 2021-03-23

**Authors:** Jie Li, Xu Han, Yan Gu, Jixiang Wu, Jianxiang Song, Zhan Shi, Huiwen Chang, Ming Liu, Yajun Zhang

**Affiliations:** ^1^The First School of Clinical Medicine, Nanjing Medical University, Nanjing, China; ^2^Department of Cardiothoracic Surgery, Yancheng Third People’s Hospital, The Sixth Affiliated Hospital of Nantong University, Yancheng, China; ^3^The Yancheng School of Clinical Medicine of Nanjing Medical University, Yancheng, China

**Keywords:** ESCC, lncMTX2-6, cell cycle, miR-574-5p, SMAD4

## Abstract

Esophageal squamous cell carcinoma (ESCC) has been one of the key causes of cancer deaths worldwide. It has been found that long non-coding RNA (lncRNA) is related to the generation and progression of various cancers (including ESCC). However, there are still many lncRNAs related to ESCC whose functions and molecular mechanisms have not been clearly elucidated. In this study, we first reported that lncRNA MTX2-6 was significantly downregulated in ESCC tissues and cell lines. The decreased expression of MTX2-6 is closely related to larger tumor and worse prognosis of ESCC patients. Through a series of functional experiments, we detected that overexpressed MTX2-6 inhibited cell proliferation and promoted cell apoptosis of ESCC *in vitro* and *in vivo*. Further studies showed that MTX2-6 exerts as a competing endogenous RNA (ceRNA) by binding miR-574-5p and elevates the expression of SMAD4 in ESCC. In summary, our results clarify the tumor suppressor roles of MTX2-6/miR-574-5p/SMAD4 axis in the progression of ESCC and provide emerging therapeutic targets for ESCC patients.

## Introduction

The incidence and mortality of esophageal cancer rank sixth and seventh among all malignancies in 2018, respectively. At least 570,000 diagnosed esophageal cancer patients and 500,000 deaths evaluated to occur worldwide (Bray et al., 2018). Esophageal cancer mainly includes two pathological types, squamous cell carcinoma and adenocarcinoma. Esophageal squamous cell carcinoma (ESCC) occupies about 90% of all esophageal cancer in China (Liang et al., 2017). Although considerable advances (radical surgery, neoadjuvant treatment, radiotherapy, immunotherapy, and chemotherapy) have been applied to the treatment of ESCC patients, the 5-year survival rate is still just about 30% (Okines et al., 2010; Abnet et al., 2018; Reichenbach et al., 2019). Therefore, revealing the mechanism of pathogenesis and progression is critical to improve the prognosis of ESCC patients.

Long non-coding RNAs (lncRNAs), which are absent of protein-coding ability, affect the biological behaviors (tumor growth, metastasis, chemotherapy resistance, and cancer stemness) of tumors via regulating target genes at epigenetic, transcriptional, and posttranscriptional levels (Mercer and Mattick, 2013; Yao et al., 2019; Yang et al., 2020). Numerous studies have substantiated the vital role of lncRNAs in initiation and progression of malignancies, especially the robust potentialities of lncRNAs in early diagnosis, treated targets, and prognostic biomarkers of ESCC (Chen et al., 2015; Hu et al., 2015; Tong et al., 2015; Tian et al., 2020). Further research presented that lncRNAs can significantly afflict the process of tumors through the specific mechanism of competing endogenous RNAs (ceRNAs) (Sun and Zhang, 2019; Liu et al., 2020; Zhang et al., 2020). Therefore, probing and elucidating the mechanism of the emerging ceRNAs of lncRNAs can provide an insight view of ESCC.

In this study, we found lncRNA MTX2-6 closely related to the clinical characteristics of ESCC. The MTX2-6 significantly decreased in ESCC tissues and cell lines, and the lower MTX2-6 was related to the larger tumors and poorer prognosis of patients. Importantly, the specific functions of MTX2-6 in ESCC have not been excavated yet. Through a series of functional experiments *in vitro* and *in vivo*, we found that high expression of MTX2-6 inhibited cell proliferation and promoted cell apoptosis of ESCC. With profound studies, we found that MTX2-6 regulated the expression of the target gene SMAD4 by competitively binding to miR-143-3p to exert ceRNAs function, thereby affecting the process of ESCC. In summary, our study clarified the tumor suppressor roles of MTX2-6/miR-574-5p/SMAD4 axis in ESCC, which provided prognostic markers and promising therapeutic targets for ESCC patients.

## Materials and Methods

### Clinical Specimens and Cell Culture

A total of 80 paired ESCC tissues and matched adjacent tissues were obtained from the patients who were diagnosed as ESCC by histopathological examination in Department of Cardiothoracic Surgery, Yancheng Third People’s Hospital, The Sixth Affiliated Hospital of Nantong University. Resected tumors were frozen in liquid nitrogen immediately and stored at −80°C. This study was approved by the Ethics Committee of the Sixth Affiliated Hospital of Nantong University. Patients have signed informed consent before specimens’ collection.

Esophageal squamous cell carcinoma cell lines (KYSE150, TE-1, TE-10, EC-1, and EC-109) and the normal esophageal epithelial cell (NE1) were purchased from the Cell Bank of Type Culture Collection of the Chinese Academy of Sciences (Shanghai, China). All cells were maintained in RPMI-1640 medium (Gibco, United States) supplemented with 10% foetal bovine serum (Winsent, QC, Canada) at 37°C in a 5% CO_2_ incubator.

### qRT-PCR and miRNA RT-PCR

Both qRT-PCR and miRNA RT-PCR methods referred to a previously published paper (Li et al., 2020). Supplementary Table 1 lists the primers for qRT-PCR of lncMTX2-6, β-actin, U6, SMAD4, miR-574-5p, and miR-1285-3p, the primers for RT-PCR of miR-574-5p, miR-1285-3p, and U6. The results were presented through relative quantification by the 2^–ΔΔCT^ method.

### Cell Transfection

The plasmids of overexpressed SMAD4 and negative control (NC) were designed and synthesized by GenePharma (Shanghai, China). The MTX2-6, miR-574-5p-mimics, miR-574-5p-inhibitor, and the corresponding NC used lentivirus to stably transfect with cells. Lipofectamine 3000 was used for the transfection of lentivirus and plasmids. The transfection efficiency was confirmed through qRT-PCR.

### Fluorescent *in situ* Hybridization

The procedures were consistent with the description in a previous publication (Yang et al., 2020). The probe of lncMTX2-6 and fluorescent *in situ* hybridization (FISH) Kit were designed and obtained from RiboBio (Guangzhou, China).

### Western Blot

Procedures were as described previously (Li et al., 2020). The primary antibody information is listed below: SMAD4 (Abcam, ab110175), β-actin (Abcam, ab6276), cyclin-dependent kinase 4 (CDK4) (Abcam, ab193968), cyclin D1 (Abcam, ab40754), Bcl-2 (Abcam, ab182858), Bax (Abcam, ab263897), and Caspase-3 (Abcam, ab32351).

### 5-Ethynyl-2’-Deoxyuridine Assay

The procedures were adapted from a previous publication (Zhang et al., 2018). Images were photographed by using a Nikon microscope (Nikon, Japan).

### Luciferase Reporter Assay

The procedures were performed as before described (Li et al., 2020). GeneScript (Nanjing, China) synthesized the sequences corresponding to the 3’-UTR of MTX2-6 and SMAD4 mRNA and containing the wild-type or mutated miR-574-5p binding sequence.

### RNA Immunoprecipitation

RNA Immunoprecipitation (RIP) assay was conducted as previously described (Zhang et al., 2018). The EZ-Magna RIP RNA Binding Protein Immunoprecipitation Kit (Millipore, United States) was used to conduct RIP assay. Precipitate was digested, and the co-immunoprecipitated RNA was isolated for PCR.

### Cell Proliferation Assay

After the cells were supplemented with Cell Counting Kit-8 (CCK-8) (Beyotime, Shanghai, China) for 120 min, wavelength of 450 nm was selected to detect the absorbency of cells.

For colony formation assay, the procedures were adopted from Zhang et al. (2018).

### Immunohistochemistry

Immunohistochemistry (IHC) was conducted as previously described (Li et al., 2020). IHC scores were calculated of stain-intensity scores adding scores of staining percentage. Detailed grading standards can refer to Li et al. (2020).

### Cell Cycle and Apoptosis Analysis

The cells were fixed with 75% ethanol at −20°C for at least 12 h. Subsequently, the cells stained with DNA staining dye (Beyotime, Shanghai, China) at room temperature avoiding light for 30 min. The percentage of cells was analyzed using BD FACSCanto II.

Cells were pretreated with 4 μmol H_2_O_2_ for 2 h before adding the apoptotic reagents. The treated cells were lucifugally incubated with 300 μl binding buffer, including 3 μl Annexin V-FITC and 3 μl PI, for 20 min. BD FACSCanto II was used to analyze the cells.

### Tumor Xenograft in Nude Mice Model

A 4-week-old BALB/C nude mice were used for the xenograft model. Stably transfected with MTX2-6, NC, MTX2-6 + Ctl, and MTX2-6 + miR-574-5p cells were injected subcutaneously of mice, respectively. The tumor volumes and weights were measured every week. After 21 days, the xenograft tumors were dissected. All animal experiments were approved by the Committee on the Ethics of Animal Experiments of Nantong University.

### Terminal Deoxynucleotidyl Transferase dUTP Nick-End Labelling Assay

*In situ* Cell Death Detection Kit (Roche, Germany) was used to perform the transferase dUTP nick-end labelling (TUNEL) assay by following manufacturer’s protocols. Antigen retrieval was performed using hot 0.1 M citrate buffer (pH 6.0) and incubation with the TUNEL reaction mixture for 1 h at 37°C. The apoptotic cells were analyzed using a fluorescence microscope (Leica, Germany).

### Statistical Analysis

The data were presented as mean ± standard deviation. The experiments were performed thrice independently. Statistical significances were determined using chi-square test, Student’s *t*-test and two-way ANOVA. Kaplan–Meier was used to compare ESCC patients’ prognosis. SPSS and GraphPad Prism analyzed the data, and *p*-value < 0.05 was set as significance threshold.

## Results

### MTX2-6 Is Downregulated in ESCC and Related With Adverse Clinical Features

**FIGURE 1:**
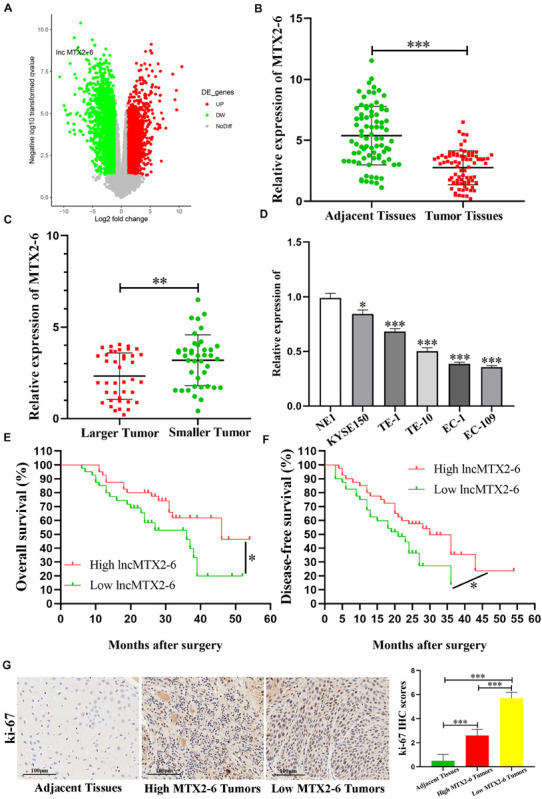
MTX2-6 is decreased in ESCC and associated with prognosis. **(A)** Volcano plot analysis of MTX2-6 expression in ESCC from the GEO database. **(B)** MTX2-6 expression in 80 paired ESCC tissues and adjacent normal tissues. **(C)** MTX2-6 expression in different tumor size of ESCC. **(D)** MTX2-6 expression in ESCC cell lines and normal cell NE1. **(E)** Kaplan–Meier analysis of overall survival (OS) curves according to MTX2-6 expression levels. **(F)** Kaplan–Meier analysis of disease-free survival (DFS) curves according to MTX2-6 expression levels. **(G)** The IHC of ki-67 in the adjacent tissues, high expression MTX2-6 of tumors, and low expression MTX2-6 of tumors. ^∗^*p* < 0.05, ^∗∗^*p* < 0.01, ^∗∗∗^*p* < 0.001.

The expression of MTX2-6 was significantly downregulated in ESCC samples than in adjacent tissues from GEO database (Figure 1A). Moreover, the decreased expression of MTX2-6 was verified in 80 ESCC tissues and 80 corresponding adjacent tissues (Figure 1B). Then, we divided the patients into two groups through the median value of tumor size. The results indicated that the expression of MTX2-6 was lower in larger tumors than that in smaller tumors (Figure 1C). Then, we detected the expression of MTX2-6 in ESCC cell lines and NE1. As expected, the expression of MTX2-6 in NE1 cells was higher than that in all ESCC cells (Figure 1D). We collected and analyzed the pathological and follow-up data of 80 ESCC patients to explore the correlation between MTX2-6 and clinical features. The results showed that the overall survival (OS) and disease-free survival (DFS) of ESCC patients with higher MTX2-6 expression were better than those of patients with lower MTX2-6 expression (Figures 1E,F). The results of ki-67 of IHC indicated that the low expression MTX2-6 tumors grew faster than the adjacent tissues or high expression of MTX2-6 tumors (Figure 1G). In addition, the expression of MTX2-6 was inversely related to the tumor size of ESCC (Table 1).

**TABLE 1:** Relevance analysis of lncMTX2-6 and miR-574-5p expression in ESCC patients.

**Characteristics**	**lncMTX2-6**		***P*-value**	**miR-574-5p**		***P*-value**
	**High**	**Low**		**High**	**Low**	
All Cases	40	40		40	40	
Age (years)						
<60	18	16	0.6507	15	19	0.3658
≥60	22	24		25	21	
Gender						
Male	23	22	0.8213	21	24	0.4990
Female	17	18		19	16	
Tumor size (cm)						
<4	28	16	0.007**	15	29	0.00165**
≥4	12	24		25	11	
T stage						
I+II	27	15	0.00722**	14	28	0.00172**
III+IV	13	25		26	12	
Lymph node metastasis						
No	17	14	0.4912	13	18	0.2511
Yes	23	26		27	22	

**P < 0.01.

### Upregulated MTX2-6 Represses ESCC Cell Proliferation *in vitro*

**FIGURE 2:**
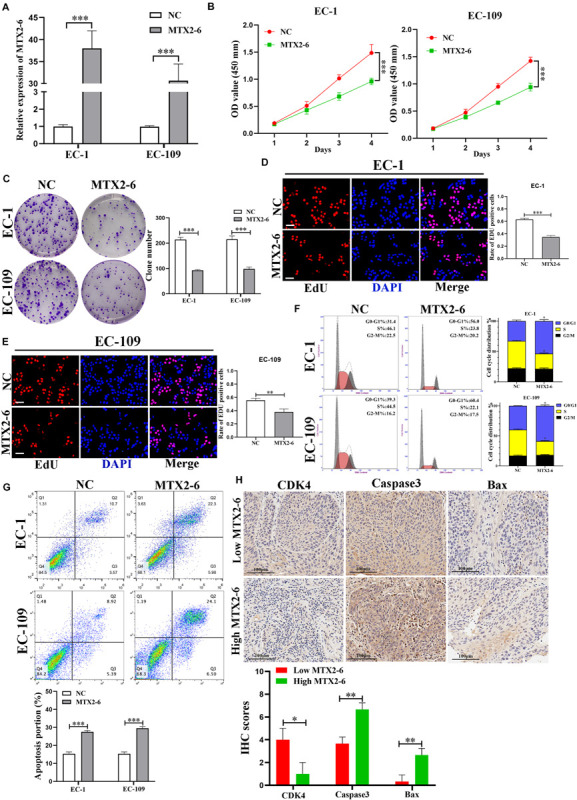
Overexpressed MTX2-6 inhibits ESCC cell proliferation *in vitro*. **(A)** qRT-PCR analysis of MTX2-6 in EC-1 and EC-109 cells treated with LV-MTX2-6. **(B)** CCK-8 assay was applied to detect cell viability. **(C–E)** Colony formation assays and the Edu assays were conducted to measure cell proliferation ability in EC-1 and EC-109 cells (scale bar: 100 μm for Edu assay). **(F)** Effects of MTX2-6 on regulating cell cycle were detected by flow cytometry. **(G)** Flow cytometry analysis of EC-1 and EC-109 cells apoptosis rates. **(H)** The IHC of CDK4, Caspase3 and Bax in the high expression MTX2-6 of tumors and low expression MTX2-6 of tumors. ^∗^*p* < 0.05, ^∗∗^*p* < 0.01, ^∗∗∗^*p* < 0.001.

We chose two ESCC cell lines EC-1 and EC-109 by the expression of MTX2-6 to conducting functional experiments. The data of Figure 2A confirmed that the expression of MTX2-6 was elevated in EC-1 and EC-109 cell lines by qRT-PCR. Overexpressed MTX2-6 decreased the cell proliferation ability of ESCC by CCK-8 assay (Figure 2B). Representative photographs of colony assay similarly showed that the upregulated MTX2-6 restrained cell growth compared to NC group of ESCC (Figure 2C). As shown in Figures 2D,E, the rates of 5-ethynyl-2′-deoxyuridine (EdU) positive cells were higher in NC groups, and MTX2-6 significantly repressed the cell proliferation through EdU assay of ESCC. In addition, elevated MTX2-6 inhibited cells from G0/G1 phase to S phase and promoted cell apoptosis of ESCC (Figures 2F,G). The results of IHC presented that the lower expression of MTX2-6 in tumors showed the higher CDK4, the lower Caspase3 and Bax than that of the higher expression of MTX2-6 tumors (Figure 2H). These results demonstrate that overexpressed MTX2-6 repressed ESCC cell proliferation, inhibited cell cycle into S phase, and promoted ESCC cell apoptosis.

### MTX2-6 Exerts on a Molecular Sponge for miR-574-5p of ESCC

**FIGURE 3:**
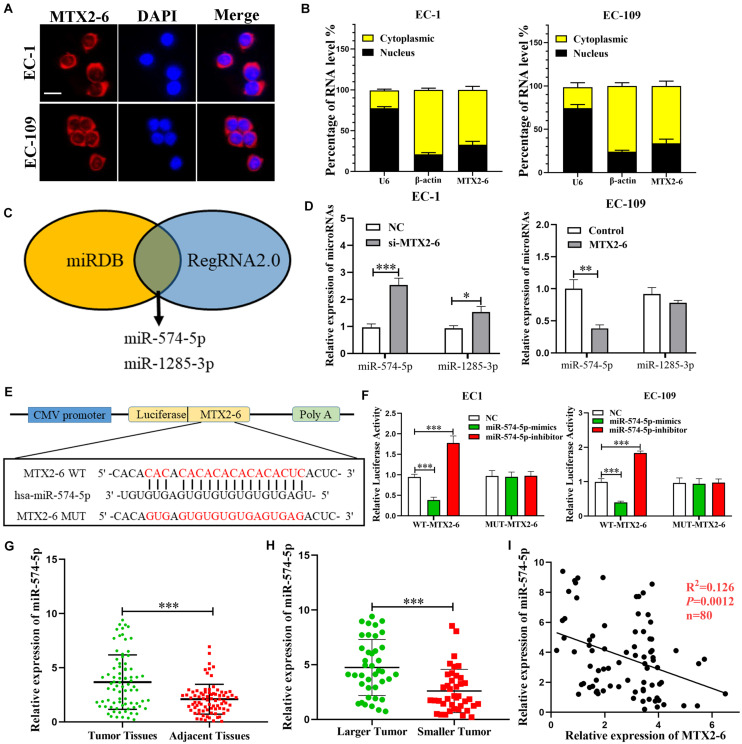
MTX2-6 acts as a molecular sponge for miR-574-5p in ESCC cells. **(A,B)** Subcellular localization of MTX2-6 in ESCC cells was analyzed by FISH and qRT-PCR (scale bar: 25 μm for FISH assay). **(C)** miRDB and RegRNA2.0 databases predict miRNAs binding to MTX2-6. **(D)** The relative expressions of miRNAs in ESCC cells after upregulation or downregulation of MTX2-6. **(E)** The predicted binding sites between MTX2-6 and miR-574-5p. **(F)** miR-574-5p mimics markedly reduced luciferase activity in MTX2-6-wild, not in MTX2-6-mut in HEK-293T cells. **(G)** Relative expression of miR-574-5p in ESCC tissues and adjacent tissues. **(H)** miR-574-5p expression in different tumor size of ESCC. **(I)** Correlation analysis of the expression of MTX2-6 and miR-574-5p in 80 ESCC tissues. ^∗^*p* < 0.05, ^∗∗^*p* < 0.01, ^∗∗∗^*p* < 0.001.

To further explore the specific mechanism of MTX2-6, we first confirmed the subcellular localization of MTX2-6 by FISH and qRT-PCR. The results presented that MTX2-6 was mainly located in the cytoplasm of ESCC cells (Figures 3A,B). Therefore, we hypothesized that MTX2-6 may act as ceRNA of microRNAs. Then, we used miRDB^1^ and RegRNA2.0^2^ databases to predict miRNAs that may interact with MTX2-6, respectively. Through the integration and analysis of these results, miR-574-5p and miR-1285-3p may exist in close interaction with MTX2-6 (Figure 3C). The level of miR-574-5p and miR-1285-3p expression increased when MTX2-6 was downregulated, but only miR-574-5p significantly decreased when MTX2-6 was upregulated by qRT-PCR (Figure 3D). Therefore, we focused on the relation between MTX2-6 and miR-574-5p. The luciferase reporter presented that elevated miR-574-5p remarkably decreased the luciferase activity of wild-type MTX2-6 compared with mutant-type MTX2-6 (Figures 3E,F). Then, the levels of miR-574-5p expression in the 80 paired ESCC tissues and adjacent tissues were detected. The level of miR-574-5p expression was higher in the ESCC tissues (Figure 3G), which indicated it may exert on an oncogenic gene. In addition, the level of miR-574-5p was higher in larger tumors in comparison with that in smaller tumors (Figure 3H). It was a negative correlation between the level of MTX2-6 expression and miR-574-5p in 80 paired ESCC specimens (Figure 3I). Abovementioned data indicated that there is a ceRNA between MTX2-6 and miR-143-3p.

### The Effects of MTX2-6 on ESCC Cells Are Regulated by miR-574-5p

**FIGURE 4:**
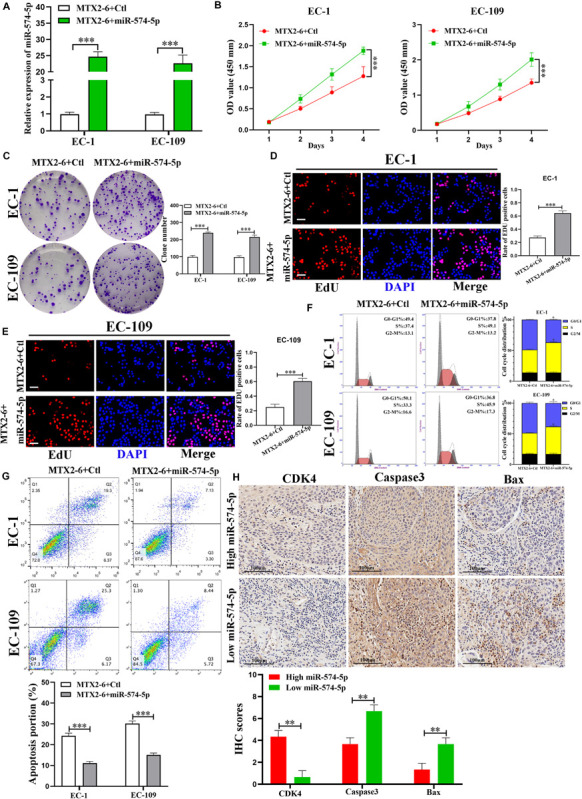
The effects of MTX2-6 on ESCC cells are mediated by miR-574-5p. **(A)** Expression of miR-574-5p was verified in co-transfected ESCC cell lines by qRT-PCR. **(B–E)** CCK-8 assays, colony formation assays, and the Edu assays confirmed that overexpressed miR-574-5p abrogated the effects of upregulated MTX2-6 on proliferation of ESCC cells (scale bar: 100 μm for Edu assay). **(F)** miR-574-5p restoration promoted cell cycle from G0/G1 phase stepped to S phase of MTX2-6-overexpressing of ESCC cells. **(G)** miR-574-5p restoration inhibited cell apoptosis of MTX2-6-overexpressing of ESCC cells. **(H)** The IHC of CDK4, Caspase3, and Bax in the high expression miR-574-5p of tumors and low expression miR-574-5p of tumors. ^∗^*p* < 0.05, ^∗∗^*p* < 0.01, ^∗∗∗^*p* < 0.001.

The expression of miR-574-5p elevated in co-transfected cell lines (EC-1 and EC-109) by qRT-PCR (Figure 4A). Overexpressed miR-574-5p reversed the inhibited effects of upregulated MTX2-6 on cell proliferation ability of ESCC by CCK-8, colony formation, and EdU assays (Figures 4B–E). Moreover, elevated miR-574-5p facilitated cells from G0/G1 phase to S phase and inhibited cell apoptosis of ESCC (Figures 4F,G). The IHC results presented that the higher expression of miR-574-5p in tumors showed the higher CDK4, the lower Caspase3 and Bax than that of the higher miR-574-5p expression in tumors (Figure 4H). Abovementioned results suggest that the inhibition on ESCC cell growth of overexpressed MTX2-6 is mediated by miR-574-5p.

### MTX2-6 Inhibited ESCC Cell Proliferation via miR-574-5p *in vivo*

**FIGURE 5:**
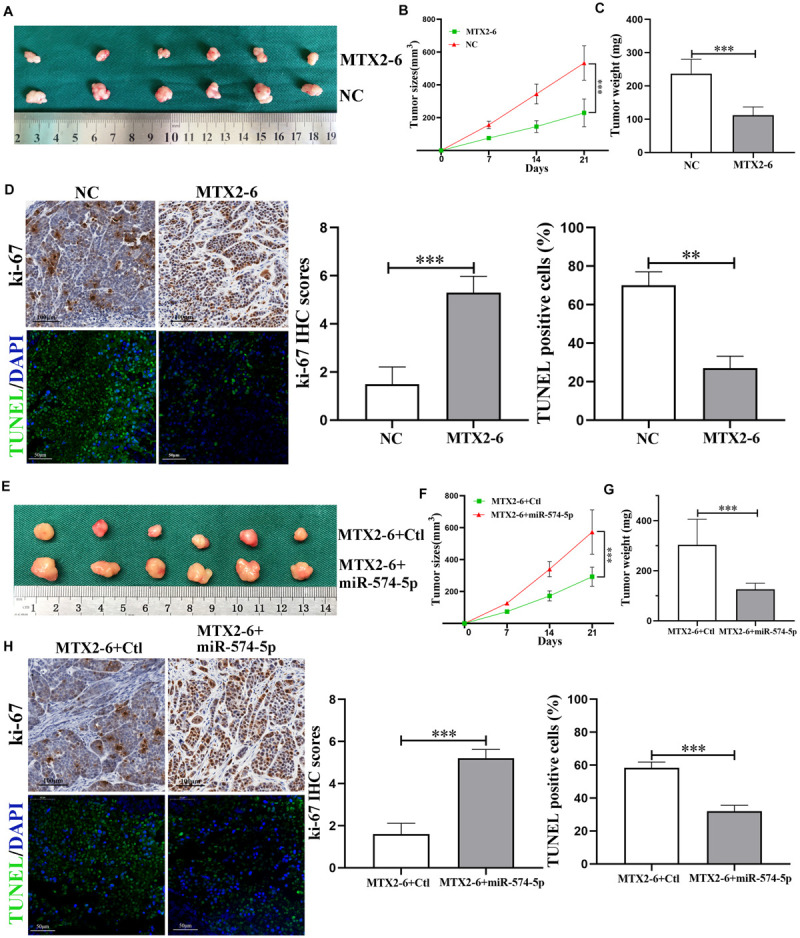
MTX2-6 inhibits cell proliferation through regulating miR-574-5p of ESCC *in vivo*. **(A)** Photograph of tumors obtained from nude mice transfected with MTX2-6 and NC. **(B,C)** Analysis of tumor size and weight in different groups. **(D)** Protein levels of Ki-67 in the tumor samples were determined by immunohistochemical. Scale bar = 100 μm. TUNEL was used to detect the apoptosis in the tumor samples. Scale bar = 50 μm. **(E)** Photograph of tumors obtained from nude mice co-transfected with MTX2-6 + Ctl and MTX2-6 + miR-574-5p. **(F,G)** Analysis of tumor size and weight in different groups. **(H)** Protein levels of Ki-67 in the tumor samples were determined by immunohistochemical. Scale bar = 100 μm. TUNEL was used to detect the apoptosis in the tumor samples. Scale bar = 50 μm. ^∗∗^*p* < 0.01, ^∗∗∗^*p* < 0.001.

To confirm the biological functions of MTX2-6 *in vivo*, the upregulated MTX2-6 cells, NC cells, co-transfected MTX2-6, and control cells and co-transfected MTX2-6 and miR-574-5p cells were subcutaneously injected to the nude mice. After 3 weeks, as shown in Figure 5A, the MTX2-6 significantly repressed the tumor growth of ESCC cells, and the tumor size and tumor weight of the MTX2-6 group were smaller and lighter than that of the NC group (Figures 5B,C), respectively. From the IHC assay of ki-67 and TUNEL assay (Figure 5D), the results indicated that overexpressed MTX2-6 can inhibit growth and promote the apoptosis of ESCC cells *in vivo*. Interestingly, overexpressed miR-574-5p reversed the inhibition on tumor growth of upregulated MTX2-6 of ESCC cells *in vivo* (Figure 5E). The results of the co-transfected MTX2-6 and miR-574-5p group presented the larger tumors and heavier tumors than that of MTX2-6 and Ctl group (Figures 5F,G). The upregulated miR-574-5p boosted the tumor growth and inhibited tumor apoptosis (Figure 5H). These results indicated that MTX2-6 restrained the adverse behaviors of ESCC via regulating the miR-574-5p *in vivo*.

### SMAD4, a Target of miR-574-5p, Is Indirectly Regulated by MTX2-6

**FIGURE 6:**
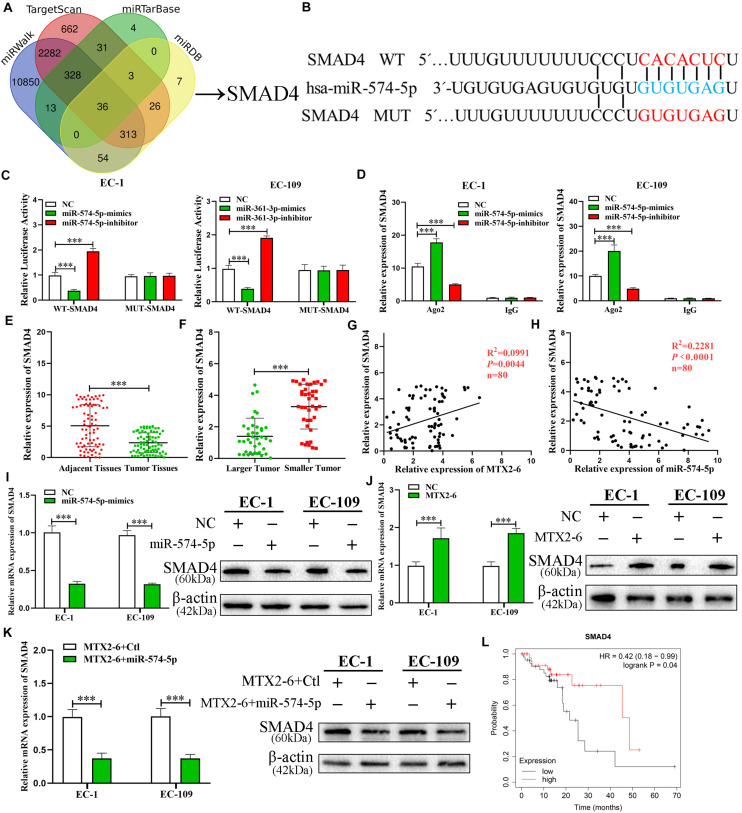
SMAD4, a target gene of miR-574-5p, is regulated by MTX2-6. **(A)** Predicted binding targets for miR-574-5p binding target via miRWalk, miRDB, TargetScan, and miRTarBase. **(B)** The predicted binding sites between SMAD4 and miR-574-5p. **(C)** Luciferase reporter assay was conducted to verify that miR-574-5p bound to the 3′-UTR region of SMAD4 directly. miR-574-5p overexpression significantly suppressed, while miR-574-5p loss increased the luciferase activity that carried wild-type (wt) but not mutant (mut) 3′-UTR of SMAD4. **(D)** RIP assays confirmed the binding status between miR-574-5p and SMAD4 in ESCC cell lines, respectively. **(E)** qRT-PCR was used to detect the ESCC expression in 72 ESCC tissues and paired adjacent tissues. **(F)** SMAD4 expression in different tumor size of ESCC. **(G,H)** Correlation analysis of the expression of MTX2-6, miR-574-5p, and SMAD4 in 80 ESCC samples. **(I)** Relative mRNA and protein levels of SMAD4 in ESCC cells transfected with miR-574-5p mimics and control groups. **(J)** Relative mRNA and protein levels of SMAD4 in ESCC cells transfected with LV-MTX2-6 and control groups. **(K)** Relative mRNA and protein levels of SMAD4 in ESCC cells co-transfected with MTX2-6 + Ctl and MTX2-6 + miR-574-5p. **(L)** Online Kaplan–Meier overall survival (OS) curves according to SMAD4 expression levels of ESCC. ^∗∗∗^*p* < 0.001.

In order to reveal the ceRNA networks between MTX2-6, miR-574-5p, and its target in ESCC, miRWalk^3^, TargetScan^4^, miRTarBase^5^, and miRDB^1^ database were utilized to seek the potential targets of miR-574-5p (Figure 6A). A conservative putative miR-574-5p bound to the 3′UTR of SMAD4 mRNA was found among the candidate genes (Figure 6B). The results of RIP assay and luciferase reporter validated this hypothesis that miR-574-5p directly bound to SMAD4 mRNA (Figures 6C,D). Then, we detected the expression of SMAD4 in the ESCC tissues and adjacent tissues. The SMAD4 significantly decreased in ESCC tissues (Figure 6E). Moreover, the level of SMAD4 expression was lower in larger tumors than that in smaller tumors (Figure 6F). Regression analysis indicated that the level of SMAD4 expression was positively correlated with MTX2-6, while negatively correlated with miR-574-5p in 80 paired tissues (Figures 6G,H). Then, the decreased expression of SMAD4 mRNA and protein was validated in the upregulated miR-574-5p cells of ESCC (Figure 6I). The SMAD4 increased along with the upregulated MTX2-6 (Figure 6J). In rescue experiments, upregulated miR-574-5p reversed the elevation of SMAD4 expression caused by upregulated MTX2-6 (Figure 6K). Furthermore, the online Kaplan–Meier analysis^6^ indicated that a high level of SMAD4 expression was connected to a better OS of ESCC patients from TCGA database (Figure 6L). These results indicated that MTX2-6 inhibits cell proliferation by competitively binding miR-574-5p to increase SMAD4 expression of ESCC.

### The Effects of SMAD4 on ESCC Cells Is Regulated by miR-574-5p

**FIGURE 7:**
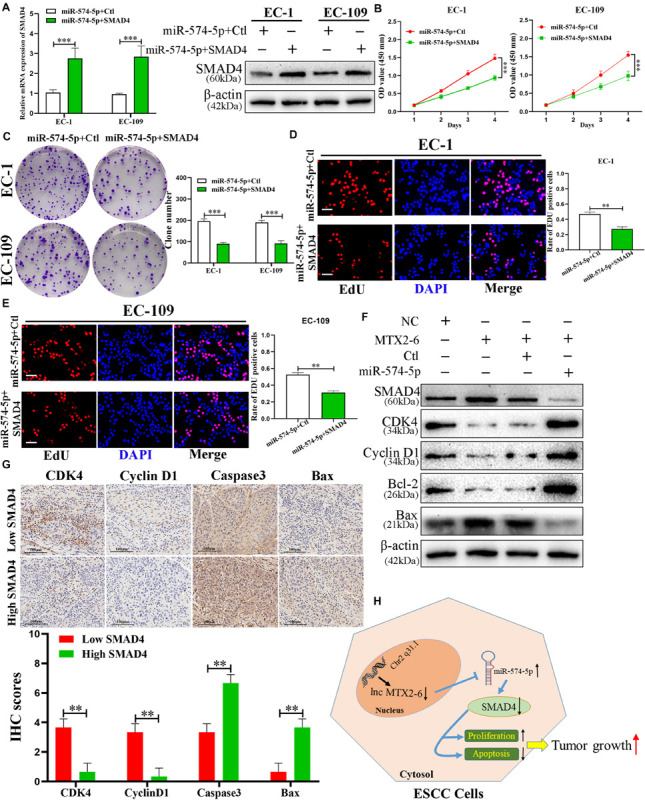
The regulation of SMAD4 on ESCC cells is mediated by miR-574-5p. **(A)** Expression of SMAD4 was confirmed by qRT-PCR and western blot in co-transfected ESCC cell lines. **(B–E)** CCK-8 assays, colony formation assays, and EdU assays were used to detect the cell proliferation after co-transfecting ESCC cells with miR-574-5p + Ctl and miR-574-5p + SMAD4 (scale bar: 100 μm for Edu assay). **(F)** Western blot analysis confirmed that MTX2-6 inhibits cell cycle process and promotes cell apoptosis through regulating miR-574-5p of ESCC. **(G)** The IHC of CDK4, Cyclin D1, Caspase3, and Bax in the high expression SMAD4 of tumors and low expression SMAD4 of tumors. **(H)** Proposed model of lnc MTX2-6 suppresses proliferation by acting as ceRNA of miR-574-5p to modulate SMAD4 in ESCC cells. ^∗∗^*p* < 0.01, ^∗∗∗^*p* < 0.001.

Preceding publications reported that SMAD4 plays as a tumor inhibitor of ESCC (Chiao et al., 1999; Natsugoe et al., 2002). MiR-574-5p + Ctl and miR-574-5p + SMAD4 were co-transfected to the cells to further study the interactions between miR-574-5p and SMAD4 of ESCC. The results of qRT-PCR and immunoblotting demonstrated that upregulated SMAD4 altered the reduction of SMAD4 mRNA and protein caused by overexpressed miR-574-5p (Figure 7A). Assays of cell proliferation detected that elevated SMAD4 inverted the effects of upregulated miR-574-5p on promotion of ESCC cell growth (Figures 7B–E). The results of Western blot further verified that upregulated MTX2-6 arrested the cell cycle from G0/G1 phrase to S phrase (CDK4 and Cyclin D1) and facilitated the cell apoptosis (Bcl-2 and Bax) via the accumulation of SMAD4 by depletion of miR-574-5p (Figure 7F). The IHC results presented that the lower expression of SMAD4 in tumors showed the higher CDK4 and Cyclin D1, the lower Caspase3 and Bax than that of the higher expression of SMAD4 tumors (Figure 7G).

## Discussion

Accumulating studies have substantiated that abnormal expression of lncRNAs is connected with the generation and progression of ESCC, and can be used as therapeutic targets and biomarkers for ESCC (Tong et al., 2015; Yao et al., 2019). This study reported for the first time that MTX2-6 was decreased in ESCC tissues and cell lines. Moreover, the larger tumors exhibited a lower level of MTX2-6 expression than the smaller tumors of ESCC. It was a positive correlation between the level of MTX2-6 expression and the prognosis of ESCC patients. All results indicated that MTX2-6 plays an inhibitive factor in ESCC.

Adverse clinical features are the main causes for the poor prognosis of ESCC patients. The increasing evidence shows that lncRNAs significantly regulate the cell cycle and apoptosis in various cancers, thereby affecting tumor growth, including ESCC (Liu et al., 2019; Fan et al., 2020; Yang et al., 2020). In this study, functional experiments proved that elevated MTX2-6 represses the growth of ESCC cells via arresting the cell cycle from G0/G1 phrase to S phrase and facilitating cell apoptosis *in vitro* and *in vivo*.

In recent years, the ceRNA theory of tumors has drawn widespread investigations. Previous studies demonstrated that lncRNAs can exert as a ceRNA of binding to the specific miRNAs to altering target genes (Liu et al., 2014; Sanchez-Mejias and Tay, 2015). FISH and qRT-PCR were used to confirm the cytoplasm localization of MTX2-6 in ESCC cells. Then, we found the putative target miRNA of MTX2-6 by using bioinformatic tools, miR-574-5p, which was reported as an oncogenic gene in ESCC (Yang et al., 2013; Han et al., 2020). The luciferase activity of Wild Type of MTX2-6, and not that of mutation, is altered by the overexpressed and downregulated of miR-574-5p. Next, the elevated level of miR-574-5p was validated in the 80 ESCC tissues in comparison with adjacent tissues. In addition, the level of miR-574-5p expression was negatively correlated with MTX2-6 expression in ESCC tissues. miR-574-5p mediated the MTX2-6 regulation of ESCC cell cycle and apoptosis. Upregulated miR-574-5p reversed the inhibition on cell growth of MTX2-6 *in vitro* and *in vivo*. The ceRNA network between MTX2-6 and miR-574-5p was gradually detected of ESCC.

Therefore, the most critical target gene in the ceRNA network was expected to excavate. Similarly, the miRWalk, TargetScan, miRTarBase, and miRDB databases were applied to seek the objects of miR-574-5p. SMAD4 was sieved to further investigate luciferase reporter assays and RIP assays, which was investigated as a tumor suppressor in ESCC (Osawa et al., 2000; Natsugoe et al., 2002). We detected that the level of SMAD4 expression decreased in 80 ESCC tissues in comparison with adjacent tissues. Higher levels of SMAD4 indicated better prognosis and clinical features of ESCC patients. Besides, the positive correlation between MTX2-6 and SMAD4 and the negative relation between SMAD4 and miR-574-5p were checked in the tissues of ESCC. Rescue experiments revealed that miR-574-5p repressed SMAD4 abundance in ESCC cells, while overexpressed SMAD4 abolished the changes of miR-574-5p-induced booming cell growth of ESCC. Thus, all results indicated that upregulated MTX2-6 arrested the cell cycle from G0/G1 phrase to S phrase (CDK4 and Cyclin D1) and facilitated the cell apoptosis (Bcl-2 and Bax) via the accumulation of SMAD4 by depletion of miR-574-5p.

To conclude, the study first confirmed that the expression of MTX2-6 decreased in both ESCC tissues and cells and that the low MTX2-6 expression was related to adverse prognosis and clinicopathological features. Moreover, MTX2-6 inhibited the cell proliferation and facilitated cell apoptosis of ESCC via the accumulation of SMAD4 by exerting as a ceRNA that sponges miR-574-5p. In conclusion, the abnormal MTX2-6 acted a crucial part in tumor growth and was an emerging biomarker and target of treatment for ESCC (Figure 7H).

## Data Availability Statement

The original contributions presented in the study are included in the article/Supplementary Material, further inquiries can be directed to the corresponding author.

## Ethics Statement

The studies involving human participants were reviewed and approved by the Ethics Committee of the Sixth Affiliated Hospital of Nantong University. The patients/participants provided their written informed consent to participate in this study. The animal study was reviewed and approved by the Committee on the Ethics of Animal Experiments of Nantong University.

## Author Contributions

JL, XH, and YZ designed the experiments. JL and XH conducted the functional experiments. XH and YG performed the Western blots. JW and JS performed the animal experiments. ZS, HC, and ML helped to refine the writing. YZ supervised the overall research. All authors approved the submitted version.

## Conflict of Interest

The authors declare that the research was conducted in the absence of any commercial or financial relationships that could be construed as a potential conflict of interest.

## Footnotes

1 http://mirdb.org/

2 http://regma2.mbc.nctu.edu.tw

3 http://mirwalk.umm.uni-heidelberg.de/

4 http://www.targetscan.org/vert_72/

5 http://mirtarbase.cuhk.edu.cn/php/index.php

6 http://ualcan.path.uab.edu/analysis.html
