# Supplementary material for: LncRNA MTX2-6 Suppresses Cell Proliferation by Acting as ceRNA of miR-574-5p to Accumulate SMAD4 in Esophageal Squamous Cell Carcinoma
Source: Front Cell Dev Biol. 2021 Mar 23;9:654746. doi: 10.3389/fcell.2021.654746 (PMC8044847; doi:10.3389/fcell.2021.654746)
Supplement: Supplementary file 1 [file Table_1.docx]

**Supplementary Table 1: The primers for qRT-PCR.**

| hsa-LncMTX2-6 | Forward Primer | CATCCTCTGGGGCAAAACTA |
| --- | --- | --- |
|  | Reverse Primer | TAGCACAGAGGGAAGGAGGA |
| hsa-β-actin | Forward Primer | CATGTACGTTGCTATCCAGGC |
|  | Reverse Primer | CTCCTTAATGTCACGCACGAT |
| hsa-U6 | Forward Primer | CTCGCTTCGGCAGCACA |
|  | Reverse Primer | AACGCTTCACGAATTTGCGT |
| hsa-SMAD4 | Forward Primer | CTCATGTGATCTATGCCCGTC |
|  | Reverse Primer | AGGTGATACAACTCGTTCGTAGT |
| hsa-miR-574-5p | Forward Primer | CGGGCTGAGTGTGTGTGTGTG |
|  | Reverse Primer | CAGCCACAAAAGAGCACAAT |
| hsa-miR-1285-3p | Forward Primer | CGGGCTCTGGGCAACAAAGT |
|  | Reverse Primer | CAGCCACAAAAGAGCACAAT |

**The primers for RT-PCR**

| hsa-miR-574-5p | CCTGTTGTCTCCAGCCACAAAAGAGCACAATATTTCAGGAGACAACAGGACACACT |
| --- | --- |
| hsa-miR-1285-3p | CCTGTTGTCTCCAGCCACAAAAGAGCACAATATTTCAGGAGACAACAGGAGGTCTC |
| hsa-U6 | AACGCTTCACGAATTTGCGT |
